# The role of configurational disorder on plastic and dynamic deformation in Cu_64_Zr_36_ metallic glasses: A molecular dynamics analysis

**DOI:** 10.1038/srep40969

**Published:** 2017-01-19

**Authors:** S. D. Feng, K. C. Chan, S. H. Chen, L. Zhao, R. P. Liu

**Affiliations:** 1 Advanced Manufacturing Technology Research Centre, Department of Industrial and Systems Engineering, The Hong Kong Polytechnic University, Hong Kong; 2 State Key Laboratory of Metastable Materials Science and Technology, Yanshan University, Qinhuangdao 066004, China

## Abstract

The varying degrees of configurational disorder in metallic glasses are investigated quantitatively by molecular dynamics studies. A parameter, the quasi-nearest atom, is used to characterize the configurational disorder in metallic glasses. Our observations suggest configurational disorder play a role in structural heterogeneity, plasticity and dynamic relaxations in metallic glasses. The broad configurational disorder regions distribution is the indicator of abundant potential deformation units and relaxations. Plastic flow, as well as relaxation, is believed to start at configurational disorder regions. The width of the shear bands and dynamic relaxations can then be regulated by the degree of configurational disorder regions in metallic glasses.

Metallic glasses (MGs), inheriting the disordered structural features of liquids^1^, have a series of heterogeneous atomic configurations^2^. Their atoms are arranged in a random way, which also contains some short and medium range order^3^. This is completely different from crystalline counterparts that have long range order in space. Our previous study show that the amorphous structure of an MG can be depicted as a nano-scale composite, consisting of densely packed clusters surrounded by low density loosely packed regions^4^. When MGs are stressed, shear translations could primarily appear in these loosely packed interconnecting regions. Chen *et al*. demonstrated that the structure of MGs may be fractal, of dimension between fractal short-range order and long-range order, by combining experiments and molecular dynamics simulations^5^. Molecular dynamics simulations showed that an appreciable number of icosahedra could constitute medium-range order in MGs^6^. Experimentally, Hirata *et al*. recently confirmed short-range order clusters and their assemblies in MGs by nano-beam electron diffraction^7^. In addition, a number of studies have shown that the atomic structure of an MG is not uniform^8^.

Because of the specific atomic structure, those “defects” in MGs and the interaction between the defects are different from those of the crystal, and leads to different mechanical behavior^9^,^10^. The plastic deformation of the crystalline alloy mainly depends on the dislocation motion, while the plastic deformation in MGs is considered to have two mechanisms at present: shear transition zone (STZ)^11^ and free volume^12^. At present, there has been a wide range of research to prove the existence of the deformation unit via computer simulation^13^,^14^,^15^ and experimental methods^16^,^17^. For example, recent experiments report that there are even observable variations in the local modulus^18^ and local viscoelasticity^19^ at the nanometer scale. These are obviously related to the internal structure of MGs, in which local structure varies significantly on the nanometer scale. However, it is still disputable as to how the heterogeneous atomic structure affects the plastic deformation of MGs.

Johari *et al*. pointed that the relaxation behavior of MGs contains two main processes: local and reversible *β* relaxation, and large-scale and irreversible α relaxation^20^. Dynamic heterogeneity in MGs, like *β* relaxation, also are affected by the nano-scale heterogeneity, which is demonstrated through molecular dynamic simulations^21^,^22^ and experimental phenomena^23^,^24^. Yu *et al*. found that the activation energy of *β* relaxation is almost identical to the potential barrier of STZs^25^, they concluded that plastic deformation and relaxation in MGs may have a similar microstructural origin through correlating the STZs and *β* relaxation to the microstructure. Jiao *et al*. found that there is a close relationship between the deformation units, dynamic relaxation behavior and structural heterogeneities^26^,^27^. Nevertheless, how the atomic structure affects the flow resistance of STZs and dynamic relaxations under different conditions is still not clear, giving to the complexity in studying the STZ and *β* relaxation, and their relationship to the atomic structure.

All in all, the nano-scale heterogeneity plays an important role in the plastic and dynamic behavior of MGs^28^,^29^. Under stress, different atomic structures can respond to different behavior^30^. So it is urgent to establish a parameter to evaluate the degrees of order in MGs quantitatively, as well as to establish a correlation between the degrees of order and mechanical properties, across the whole range of atomic configurations. Therefore we employed a structural parameter, the quasi-nearest atom (QNA), based on the evaluation of configurational disorder^31^. Pan *et al*.^32^ have then investigated structural disorder in eight metallic glass-forming systems at various temperatures by QNA. Their results showed that the scaled distribution of the number of QNA appears to be a universal property. Relationships have been proposed to reflect the correlations between QNA, potential energy and dynamical properties. They found that the parameter of QNA is general, and can be used across different MG systems. So we adopted QNA to assess the varying degrees of configurational disorder in MGs. Plastic flow, as well as β relaxation, is believed to start at configurational disorder regions. The results show that the number of QNAs and the yield strength has a negative linear correlation, and the number of QNAs and elastic modulus has also a negative linear correlation. These are helpful in understanding the structural origin of the plastic and dynamic deformations.

## Results and Discussion

### The temperature dependence of average *N*_*Q*_

**Figure 1:**
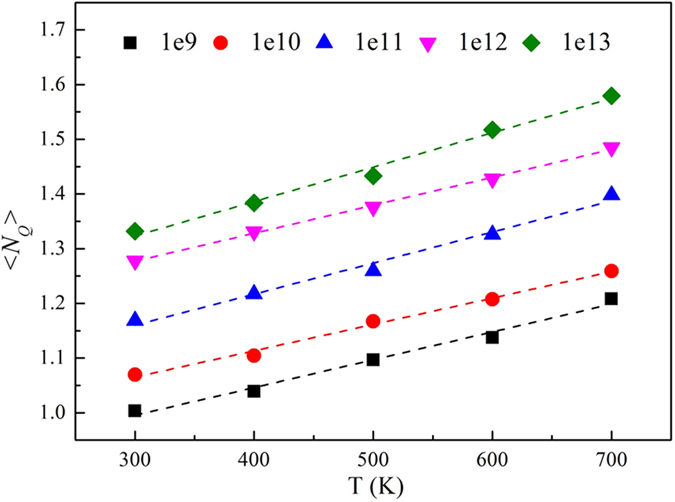
The temperature dependence of <NQ> in Cu64Zr36 MG cooled by different cooling rates.

The ability of an atom to move is related to the extent of its binding to the other atoms around it. So the number of QNAs (*N*_*Q*_) can reflect atomic mobility. In Fig. 1, the average *N*_*Q*_ (<*N*_*Q*_>) increases when the temperature increases, suggesting that the regions of configurational disorder in MGs increase. Meanwhile, with increase of cooling rate, <*N*_*Q*_> also increases strongly. This proves that the cooling rates also make the regions of configurational disorder increase. MGs with a high temperature and cooling rate may have much looser atomic packings than those with a low temperature and cooling rate, which may lead to more prominently configurational disorder. This increasing trend is in fact consistent with recent simulations in which a high temperature and rapid cooling rates weaken the degree of order in MGs^33^.

### The distribution of *N*_*Q*_ for the atoms surrounded Cu and Zr

**Figure 2:**
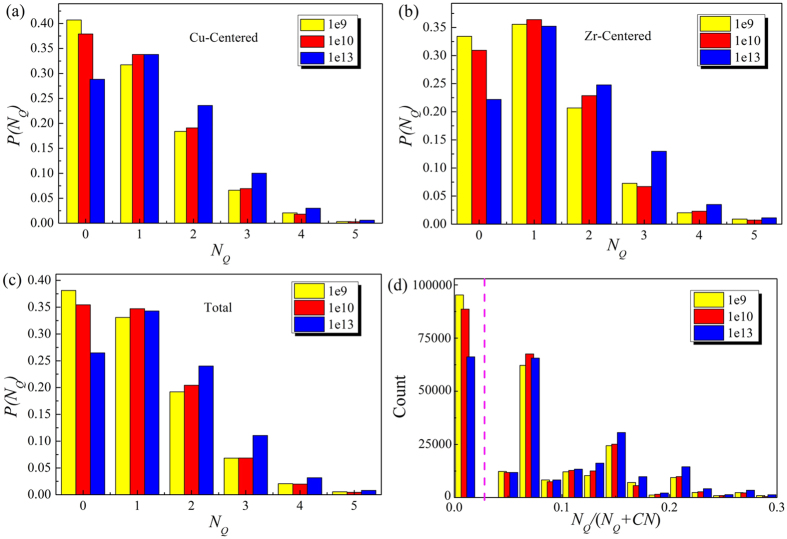
The distribution of *N*_*Q*_ for the atoms surrounded (**a**) Cu, (**b**) Zr in Cu_64_Zr_36_ MG, respectively. The distribution of (**c)** *N*_*Q*_ and (**d**) the ratio [*N*_*Q*_/(*N*_*Q*_ + *CN*)] in the Cu_64_Zr_36_ MG.

Further, to study the constitution of configurational disorder, the distribution of *N*_*Q*_ for the atoms that surrounded Cu and Zr in the Cu_64_Zr_36_ MG was investigated. As shown in Fig. 2(a), with the increase of cooling rate, the atoms that surrounded Cu with *N*_*Q*_ = 0 decrease, while the atoms surrounded Cu with *N*_*Q*_ > 0 gradually increase. This change trend is basically same with the atoms around Zr, as shown in Fig. 2(b). At the same time, their change trends are basically similar to the atoms in the entire MG, as shown in Fig. 2(c), which means that the distribution of *N*_*Q*_ barely depends on the element type. Based on *N*_*Q*_, we propose a new parameter *θ*:


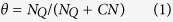


where *CN* is the coordination number. If *N*_*Q*_ is equivalent to 0, *θ* is 0, indicating configurational order. As shown in Fig. 2(d), the atoms with *θ* = 0 occupied the highest proportion, suggesting the regions of configurational order in MG represent the main body, while the regions of configurational disorder are the fillings.

### Correlation between the yield stress and averaged *N*_*Q*_

**Figure 3:**
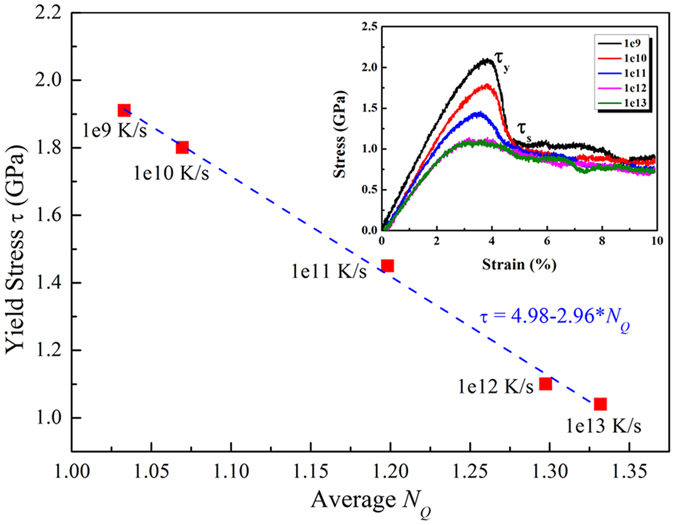
Correlation between the yield stress and averaged *N*_*Q*_ of models measured with different cooling rates (CRs). The insert shows stress–strain curves of three models subjected to different CRs.

At the atomic scale, the regions of configurational disorder should have more free volume than the configurational order regions, so the atoms in the regions of configurational disorder can move or jump much more easily when stimulated, which results in the regions of configurational disorder playing an important role in the mechanical response^34^. To understand how configurational disorder is associated with the plastic deformation, the yield strengths of MGs with different degrees of configurational disorder were investigated. Due to the inherent limitations in molecular dynamic simulation where the time scale in MD simulation is much shorter than experimental time scale, only the deformation of metallic glasses at a low temperature of 50 K was simulated. The computer quenched glass structure after rapid cooling is usually more prone to thermal activations than real-world MGs that have experienced much more extended relaxation. In fact, this is a great challenge in the computational community^21^. Therefore, in this work, the low temperature (50 K) was used to highlight the material responses upon mechanical activation. As shown in Fig. 3, the results indicate that with increasing regions of configurational disorder, the yield stress becomes lower and lower. An approximate linear relationship of [τ = 4.98 − 2.96*N_*Q*_] was obtained. By adjusting the regions of configurational disorder, we can change the yield strength. The insert in Fig. 3 shows the stress–strain curves of the MGs with different cooling rates (CRs). When the stress exceeds the yield strength (*τ*_*y*_), shear transformation zones begin to evolve into shear bands^35^. When the strain is about 6%, the stress tends to be at a steady state (*τ*_*s*_), corresponding to the strength of the shear bands. Recent studies showed that the value of Δ*τ* (Δ*τ* = *τ*_*y*_ − *τ*_*s*_) determines the strain localization tendency, thus affecting the plasticity of MGs^36^. The smaller the value of Δ*τ*, the better the plasticity of the MG.

### Correlation between the width of shear band and averaged *N*_*Q*_

**Figure 4:**
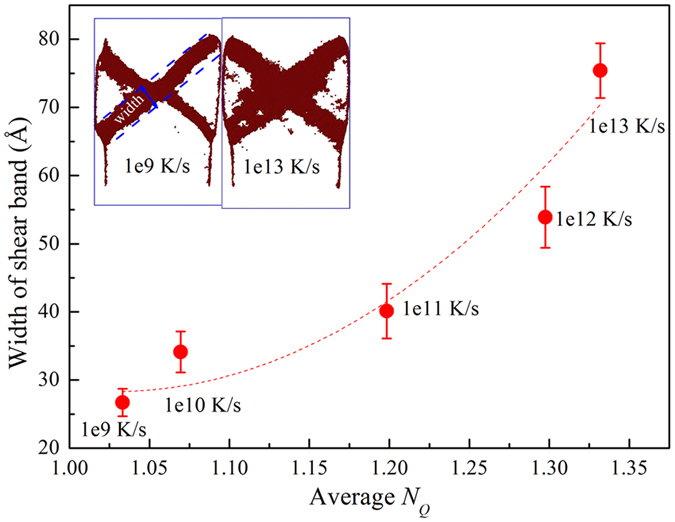
Correlation between the width of shear band and averaged *N*_*Q*_ of models measured with different CRs when the total strain is 10%. The inserts show local shear strain of models cooled by 1e9 K/s and 1e13 K/s.

The above results indicate that the regions of configurational disorder have a strong relation with yield strength and plasticity. These regions are similar to the trigger switches. Through changing the amount or distribution of these regions, the plastic performance of the MGs can be adjusted. To study the origin of varying plastic performance, the effects of these regions on the width of the shear bands were investigated, as shown in Fig. 4. In the simulation, we define that the atoms constitute the shear band region when the local shear strain is higher than 20%^37^, as shown in the insert of Fig. 4. The correlation between the width of the shear band and the averaged *N*_*Q*_ of MGs measured with different CRs was quantified. The width of the shear bands were measured on the primary shear band along the direction of 45°, as shown in the inset in Fig. 4, and the avarage value of 20 initial configurations was taken to plot the correlations. The width of MGs cooled at 1e9 K/s is 26.7 Å, while that of MGs cooled at 1e13 K/s is 75.4 Å. It is found that with the increase of <*N*_*Q*_>, i.e. the regions of configurational disorder increases, the shear bands broaden nearly two times, which further explains the reasons that plasticity becomes better in MGs cooled at higher CRs.

This is due to an MG with a greater cooling rate having more configurational disorder regions. STZs occur preferentially in configurational disorder regions with lower critical shear stress. With more regions of configurational disorder, the result is the formation of more STZs. During the elastic stage, these STZs are stable because they are embedded in configurational order regions. So the overall behavior seems elastic, but locally, plastic events are already activated. Regions with configurational disorder are the first to overcome the activation barrier by the external stresses, and once the MG yielding was loaded these STZs penetrate and form SBs, resulting in the wider SBs. These configurational disorder regions may be precursors of the “carriers” or the deformation units in MGs. So the characteristics of the configurational disorder can be successful in explaining many phenomena, such as shear localization, strain hardening, and the emergence of yield in MGs. These studies also indicate that configurational disorder regions are not independent, but are correlated to each other. Nevertheless, the correlation between *N*_*Q*_ and the width of the shear bands in MGs illustrates that the configurational disorder regions can stimulate the formation of more STZs, providing guidance in the design of ductile MGs.

### Correlation between dynamic deformations and averaged *N*_*Q*_

**Figure 5:**
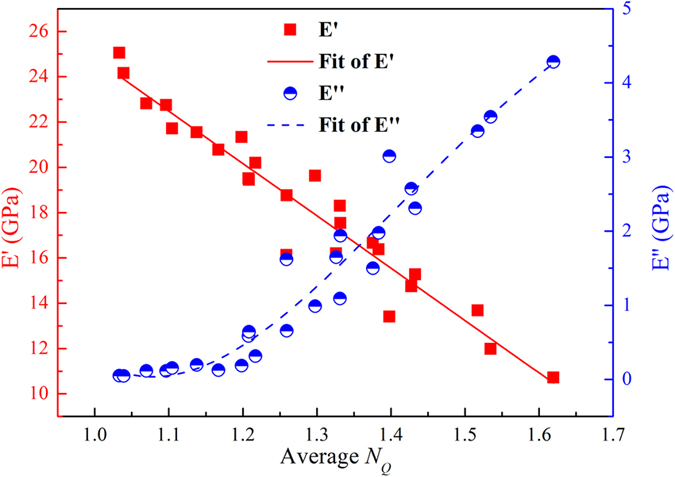
Storage modulus *E*′ and loss modulus *E*″ as functions of averaged *N*_*Q*_. In this figure, the frequency was set to 1 GHz. Temperature were 300 K, 400 K, 500 K, 600 K and 700 K. The metallic glass models were prepared at five different cooling rates: 1e9 K/s, 1e10 K/s, 1e11 K/s, 1e12 K/s and 1e13 K/s.

The above results show that in an MG, the configurational disorder regions are associated with the activation and evolution processes of the deformation units. Not only are the regions of configurational disorder nucleation positions of STZs, but also may be the origin of *β* relaxation. To verify this, we did dynamic mechanical testing in MD simulations. Yu and Qiao *et al*. studied *β*-relaxations in a large number of La-, Pd-, Zr-, Cu- and Ti-based MGs via dynamic mechanical analysis^38^,^39^. The above research findings suggest that the structural and dynamic heterogeneities in MGs have strong relations. To quantify the relation, the storage modulus *E*′ and loss modulus *E*″ as functions of <*N*_*Q*_> are reported, as shown in Fig. 5. The frequency was set to 1 GHz. Temperature were 300 K, 400 K, 500 K, 600 K and 700 K. The metallic glass models were prepared at five different cooling rates: 1e9 K/s, 1e10 K/s, 1e11 K/s, 1e12 K/s and 1e13 K/s. Although coordination number (CN) could reflect the packing density of corresponding regions, it cannot provide the geometric symmetry of these regions. It is worth to note that the atoms in dense-packed icosahedral clusters have been proved to move slower than other atoms in metallic glasses^3^. However, in some metallic glasses, icosahedral clusters are absent^40^. Therefore, the icosahedrons cannot be adopted as a universal structural indicator for dynamic heterogeneity. Peng *et al*. used the fraction of the number of pentagons and the number of the nearest-neighbor atoms, *d*_5,_ to define the degree of the local fivefold symmetry, where *d*_5_ = *n*_5_/CN^13^. *n*_5_ is one of parameters in the Voronoi polyhedral index, which is expressed as <*n*_3_, *n*_4_, *n*_5_, *n*_6_>, where *n*_*i*_ denotes the number of *i*-edged faces of the Voronoi polyhedron. They found that the degree of the local fivefold symmetry is crucial for the understanding of structural relaxation and mechanical properties in metallic glasses. Recently, Pan *et al*. compared Pearson correlation coefficients for CN, *d*_5_ and *N*_*Q*_ around Cu and Zr^31^. It is found that the correlation parameters for *N*_*Q*_ around Cu and Zr both have larger absolute values than those for CN and *d*_5_. This suggests that *N*_*Q*_ can better reflect the correlation between local structure and dynamic heterogeneity, compared to CN and *d*_*5*_. Therefore, *N*_*Q*_ can better reflect the structure-property relationship in dynamic heterogeneity. As shown in Fig. 5, an approximate linear relationship can be seen, while there is nolinear relationship between <*N*_*Q*_> and *E*″. Compared to Fig. 2, the changing trend of *E*′ is consistent with that of *N*_*Q*_ = 0, while the changing trend of *E*″ is consistent with that of *N*_*Q*_ > 0. This proves that configurational order regions with *N*_*Q*_ = 0 mostly effect *E*′, corresponding to the elastic part, while the regions of configurational disorder (*N*_*Q*_ > 0) effect *E*″, corresponding to the plastic part. The combination of the configurational disorder and order regions may determine the dynamic mechanical properties. The comparisons of averaged *N*_*Q*_ and *d*_5_ for storage modulus *E*′ and loss modulus *E*″ were performed, as shown in Fig. S1 of the supplemental material. By comparing Fig. S1(a,b), it is found that there are similarities between <1 − *d*_5_> and <*N*_*Q*_>. However, the relationships among *E*′, *E*″ and <*N*_*Q*_> are stronger than those among *E*′, *E*″ and <1 − *d*_5_>. The adjusted R-squared which can compare the explanatory power of regression models is used to compare the difference between *N*_*Q*_ and *d*_*5*_. The larger the adjusted R-squared values are, the better the equations fit. The adjusted R-squared values for *d*_5_ and *N*_*Q*_ are compared, as shown in Table S1 of the supplemental material. It is found that the adjusted R-squared values for *N*_*Q*_ in *E*′ and *E*″ are larger than those for (1 − *d*_5_). Therefore, *N*_*Q*_ can better reflect the correlation between local structure and dynamic heterogeneity. Icosahedra and *d*_*5*_ mainly focus on the shape of clusters, while QNA mainly focuses on the defects of clusters.

**Figure 6:**
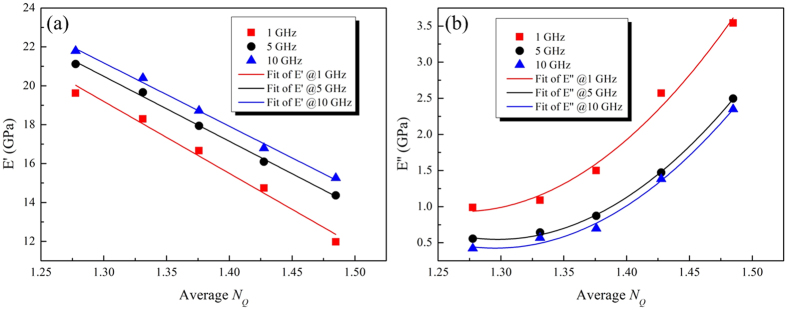
Storage modulus *E*′ and loss modulus *E*″ as functions of averaged *N*_Q_, for different frequency *f.* In this figure, the frequencies were set to 1 GHz, 5 GHz and 10 GHz. Temperature were 300 K, 400 K, 500 K, 600 K and 700 K. The metallic glass models were prepared at a cooling rate: 1e12 K/s.

In an effort to well understand the mechanical relaxations in metallic glass, it is necessary to illustrate the influence of the testing frequency on the evolution of the dynamic mechanical properties. Here, the frequencies were set to 1 GHz, 5 GHz and 10 GHz. Temperature were 300 K, 400 K, 500 K, 600 K and 700 K. The metallic glass models were prepared at a cooling rate: 1e12 K/s. Figure 6 shows the storage modulus *E*′ and loss modulus *E*″ as functions of averaged *N*_*Q*_, for different frequency *f.* With the increase of frequency, the storage modulus *E*′ becomes higher and higher, while the loss modulus *E*″ becomes lower and lower. An approximate linear relationship can be still seen, while there is nolinear relationship between <*N*_*Q*_> and *E*″. These evolutions are simply due to an increase in atomic mobility when the temperature increases or when the frequency decreases^39^. Ichitsubo *et al*. found experimentally that *β* relaxation is closely related to the soft regions of MGs^41^,^42^. The *β* relaxation increases with the increase of the soft regions. Our results are in agreement with theirs.

The formation of configurational order regions and configurational disorder regions in MGs leads to the distribution heterogeneity of defects. Since the constituent atoms in the configurational disorder regions have higher *N*_*Q*_ than those in the configurational order region, *β* relaxation, similar to the events of STZs, popularly occurs in these sites when loading shear stresses. So the appropriate configurational disorder regions with high potential energy or loosely packed cluster conformation could ameliorate *β* relaxation in MGs. In other words, these configurational disorder regions may trigger the *β* relaxations. Further, the configurational disorder regions may be the structural origin of STZs and the *β* relaxations. Yu *et al*. also found that the *β* relaxation in MGs may be the “thermal-driven events of STZs”. Nevertheless, there are differences between *β* relaxation and STZs: there is no directional flow for *β* relaxation induced by the thermal fluctuations, while there is directional flow for STZs.

## Conclusion

Based on MD simulations, the effects of configurational disorder regions constituted by atoms *N*_*Q*_ > 0 in metallic glasses were investigated. Our results show that the heterogeneity of plastic and dynamic deformation in metallic glasses arises from configurational disorder regions, and there exist close correlations between the deformation units, relaxations and quasi-nearest atoms in metallic glasses. Different numbers of QNA mark the existence of different types of configurational disordered regions in MGs. When stress is applied, the structure changes of metallic glasses are not uniform, and the plastic and dynamic deformations occur preferentially in the configurational disordered regions with strong motion ability. The measured configurational disorder may be the common origin of STZs and *β* relaxation in MGs. Regulating the degree of configurational disorder regions could adjust the heterogenicity of MGs, thereby resulting in MGs with high capability for the formation of STZs and *β* relaxation. This provides an important platform for probing atomic-level understanding of the microstructure, STZs and *β* relaxation.

## Methods

The simulations were performed by LAMMPS^43^ using an embedded atom method (EAM) potential^44^. The Cu_64_Zr_36_ MGs model, of dimensions around 22 × 4.4 × 44 nm, was filled with approximately 250,000 atoms. They were melted and equilibrated at 2000 K and 0 Pa for 1 ns with periodic boundary conditions. The models were then cooled to 50 K with different cooling rates of 0.001 K/ps, 0.01 K/ps, 0.1 K/ps, 1 K/ps and 10 K/ps. Compression tests were achieved by displacing the atoms at one end of the z axis while keeping the atoms motionless at the other end, at 50 K by applying a constant and uniaxial strain rate of of 4 × 10^7^ s^–1^. Free boundary conditions were employed along the x- and z-axis, and a periodic boundary condition was employed along the y-axis. On the other hand, dynamic mechanical spectroscopy supplied in the MD simulation was employed to obtain dynamic deformation information^45^. This method applies a sinusoidal strain *ε* = *ε*_*A*_ sin*ωt* along the z-axis, where *ω* is the period. The amplitude *ε*_*A*,_ fixed at 2.5%, was in the linear elastic regime. The resultant stress was fitted as *σ* = *σ*_*A*_sin *(ωt* + *δ)*, where *δ* is the phase difference between stress and strain. And *δ* directly reflects internal friction in MGs^44^. Storage (*E*′) and loss (*E*″) modulus values are calculated as *E*′* = σ*_*A*_/*ε*_*A*_ cos(*δ*) and *E*″* = σ*_*A*_/*ε*_*A*_ sin(*δ*), respectively.

In a metallic glass, the nearest neighbors of each atom are determined by Voronoi tessellation method^46^. In a Voronoi polyhedron, each nearest neighbor of the central atom corresponds to a face of the Voronoi polyhedron. If two Voronoi faces share an edge, the two corresponding atoms are defined as an adjacent pair of atoms. If an adjacent pair of atoms is not the nearest neighbors of each other, the two atoms are defined as a pair of QNAs. So two atoms as a pair of QNAs should meet all the following three conditions^31^: (I) they are Voronoi nearest neighbor atoms of one atom; (II) they are adjacent to each other, defined by that their corresponding Voronoi faces of the Voronoi polyhedron, centered by their common nearest neighbor, share an edge; (III) they are not the Voronoi nearest neighbor atoms of each other. Therefore, QNAs are able to represent the regions of configurational disorder.

## Additional Information

**How to cite this article**: Feng, S. D. *et al*. The role of configurational disorder on plastic and dynamic deformation in Cu_64_Zr_36_ metallic glasses: A molecular dynamics analysis. *Sci. Rep.* **7**, 40969; doi: 10.1038/srep40969 (2017).

**Publisher's note:** Springer Nature remains neutral with regard to jurisdictional claims in published maps and institutional affiliations.

## Supplementary Material

Supplemental Materials
