# Supplementary material for: The role of configurational disorder on plastic and dynamic deformation in Cu64Zr36 metallic glasses: A molecular dynamics analysis
Source: Sci Rep. 2017 Jan 19;7:40969. doi: 10.1038/srep40969 (PMC5244410; doi:10.1038/srep40969)
Supplement: Supplemental Materials [file srep40969-s1.doc]

**The role of** **configurational disorder on** **plastic and** **dynamic deformation in Cu_64_Zr_36_ metallic glasses: A** **molecular dynamics analysis**

S.D. Feng^1,2^, K.C. Chan^1,*^, S.H. Chen^1^, L. Zhao^1^, R.P. Liu^2^

^1^*Advanced Manufacturing Technology Research Centre, Department of Industrial and Systems Engineering, The Hong Kong Polytechnic University, Hong Kong.*

^2^ *State Key Laboratory of Metastable Materials Science and Technology, Yanshan University, Qinhuangdao 066004, China*

*Correspondence and requests for materials should be addressed to K.C. C ( email: kc.chan@polyu.edu.hk).


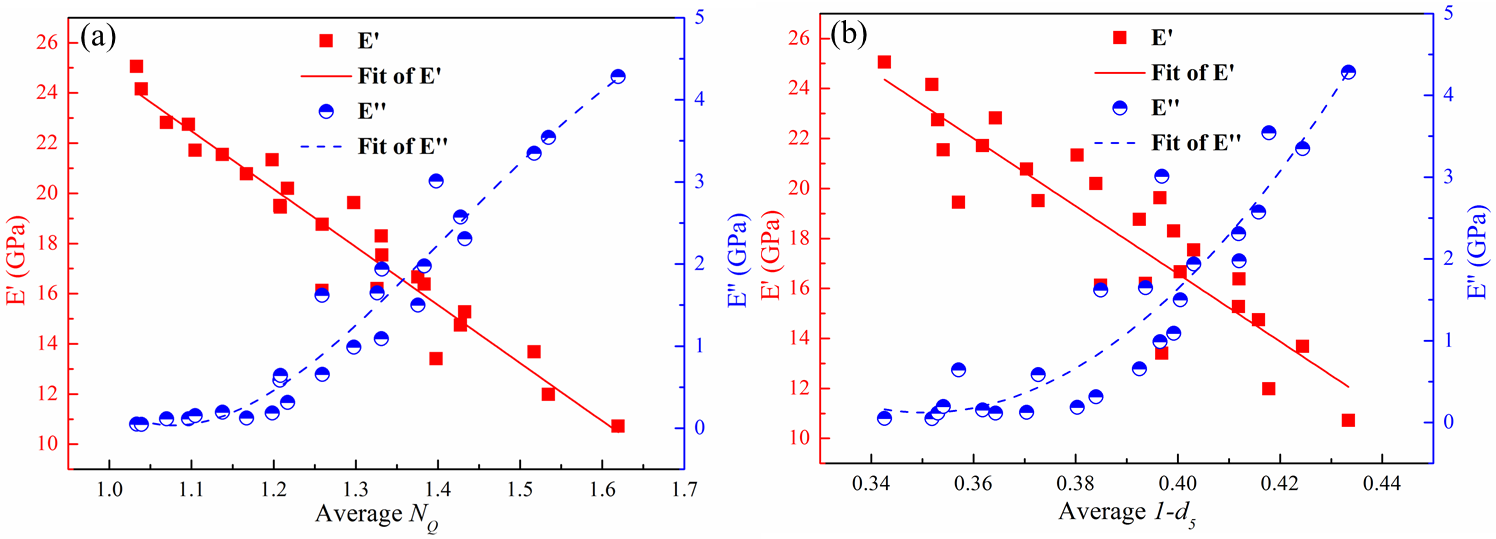


Figure S1 (a) The storage modulus *E’* and loss modulus *E’’* as functions of averaged *N_Q_*; (b) The storage modulus *E’* and loss modulus *E’’* as functions of averaged 1-*d*_5_.

Table S1 Adjusted R-squared for 1-*d*_5_ and *N_Q_* in *E’* and *E’’.*

| *Term* | *1-d5* | ***N_Q_*** |
| --- | --- | --- |
| *E’* | 0.81004 | 0.93058 |
| *E’’* | 0.83637 | 0.9349 |
